# Changes in Collective Efficacy’s Preventive Effect on Intimate Partner Violence during the COVID-19 Pandemic

**DOI:** 10.3390/ijerph191912849

**Published:** 2022-10-07

**Authors:** Toshihiko Souma, Kentaro Komura, Takashi Arai, Takahito Shimada, Yuji Kanemasa

**Affiliations:** 1 Graduate School of Humanities and Social Sciences, Hiroshima University, Hiroshima 730-0053, Japan; 2 Faculty of Humanities and Social Sciences, Hirosaki University, Aomori 036-8560, Japan; 3 Graduate School of Arts and Letters, Tohoku University, Sendai 980-8576, Japan; 4 Department of Criminology and Behavioral Science, National Research Institute of Police Science, Chiba 277-0882, Japan; 5 Faculty of Psychology, Otemon Gakuin University, Osaka 567-8502, Japan

**Keywords:** intimate partner violence, collective efficacy, COVID-19, parallel latent growth model, social distancing

## Abstract

Following the logic of studies showing that collective efficacy within neighborhoods deters intimate partner violence (IPV), the promotion of social distancing during the COVID-19 pandemic may have weakened that effect. To examine that possibility, we analyzed panel data from 318 adults in Japan regarding IPV victimization and perceived collective efficacy at four time points. A latent growth model (LGM) analysis for each measure revealed that informal social control, a subscale of collective efficacy, has declined since the pandemic began, whereas no significant changes have occurred in social cohesion and trust, another subscale of collective efficacy, and IPV victimization. Furthermore, two parallel LGM analyses revealed that although collective efficacy before the pandemic suppressed subsequent IPV victimization, changes in collective efficacy during the pandemic have been positively associated with changes in IPV. Those results suggest that collective efficacy’s protective effect on IPV is moderated by whether interactions between intimate partners and their neighbors are socially normative.

## 1. Introduction

The COVID-19 pandemic and the social distancing requirements that it has entailed may have impacted the dynamics of interpersonal relationships in society, including interactions not only between neighbors but also between partners in intimate relationships. To examine how the pandemic may have altered interactions among neighbors and within intimate relationships, we sought to determine how intimate partner violence (IPV), as a major public health challenge, has been affected by neighborhood relationships before and during the COVID-19 pandemic. In this section, we first present past research on COVID-19-induced changes in IPV, after which we discuss how collective efficacy within communities has brought about changes in IPV both before and during COVID-19.

It has been argued that the COVID-19 pandemic has increased the risk of IPV—for example, due to increased social isolation [1,2]—and some data indicate that IPV may have indeed increased since early 2020. For example, a survey of women in India revealed an increase in the number of harms that they reported, which the authors attributed both to a decrease in income and social interaction amid the pandemic and to an increase in the time spent together by victims and their perpetrators at home [3]. In the United States, a survey of residents additionally revealed that sheltering in place, a positive factor of social isolation, to prevent the spread of COVID-19 has prompted an increase in the perpetration of IPV [4]. Among other results, not only heavy drinkers, who are already predisposed to perpetrate IPV, but also light drinkers have become more likely to perpetrate IPV during the pandemic due to increased levels of stress.

Even so, other data indicate that the COVID-19 pandemic may not have altered the risk of IPV. For example, a study involving survivors of IPV before and during the pandemic revealed no particular increase in their IPV victimization [5]. Likewise, both a comparative study conducted before and after a lockdown in the Netherlands [6] and a study in Portugal conducted during the first year of the pandemic showed no statistically significant increase in IPV since the pandemic began [7]. A similarly unchanged rate of IPV was also found in a large-scale survey of a representative sample of adults in Japan conducted every 3 years by the Gender Equality Bureau, Cabinet Office [8]. In particular, the results indicated that the number of reported cases of IPV has not especially increased in recent years, even in 2020 (32.0%), which would have been partly affected by the pandemic, compared with 2014 (37.9%) and 2017 (33.2%). Data published by Japan’s National Police Agency also showed no significant increase in the number of victims of spousal physical violence or threats who consulted the police in 2020 (i.e., 82,643) or in 2021 (i.e., 83,042) compared with 2019 (i.e., 82,207) [9].

Altogether, those mixed results regarding a potentially greater risk of IPV during the COVID-19 pandemic warrant additional consideration of how the pandemic might have affected people’s risk of IPV.

### 1.1. Association between Collective Efficacy and the Incidence of IPV 

It is well-known in public health and criminology that the degree of collective efficacy in a given area affects the incidence of crime there. Defining *collective efficacy* as “social cohesion among neighbors combined with their willingness to intervene on behalf of the common good,” Sampson et al. examined the effect of social cohesion on a social process that links the structural characteristics of a neighborhood to the likelihood of crime [10] (p. 918). Because social cohesion promotes effective informal social control—that is, a community’s ability to monitor and manage social conditions that induce crime—a cohesive community that can effectively mobilize community members to regulate local crime can be understood as having a high level of collective efficacy in socially controlling crime [10,11,12].

It has often been reported that a neighborhood’s collective efficacy is associated with its incidence of both IPV victimization and perpetration. A pioneering study revealed that, in Chicago, the lower a neighborhood’s collective efficacy, the greater the rate at which women were murdered by their men partners in the neighborhood and suffered non-lethal but severe violence at the hands of their current partners [13]. The study showed that in neighborhoods with high collective efficacy, by contrast, women disclosed conflicts to others, including violence perpetrated by their partners. In other studies, neighborhood-level collective efficacy was associated with less IPV victimization among women in areas with greater socioeconomic resources available for women in general [14]. Collective efficacy has also been reported to have a suppressive effect on both IPV victimization and perpetration, especially among men [15,16]. Although other studies have not shown that effect [17], likely because they focused on highly severe IPV, Alderton et al. concluded that a large body of evidence, including from high-quality studies, nevertheless indicates that collective efficacy has a protective effect against IPV [18].

Collective efficacy can be measured as a social force in a neighborhood or as residents’ responses to that force. Whereas the former allows researchers to analyze collective efficacy’s function at the neighborhood level as an accumulation of residents’ responses, the latter allows studying its function at the individual level—that is, in respondents’ perceptions of collective efficacy in the community [19,20]. In some research, perceived collective efficacy measured at the individual level has been found to suppress IPV, as has collective efficacy measured at the neighborhood level. In Canada, data from the Quality of Neighborhood Life Survey of residents in a public housing complex in Eastern Ontario have suggested that perceived collective efficacy is associated with low IPV victimization [21]. In the United States, the results of a large study within the Fragile Families and Child Wellbeing Study have also indicated that perceived collective efficacy is negatively associated with IPV in poor neighborhoods [22]. It has additionally been reported that the greater the perception of informal social control (i.e., the tendency of neighbors to cooperate in dealing with neighborhood problems) as a subscale of collective efficacy, the lower the rate of IPV perpetration [23].

Because collective efficacy seems to suppress IPV at both the neighborhood and individual levels, we examined whether collective efficacy’s effect has changed during the COVID-19 pandemic. Our first research task was to determine whether pre-pandemic collective efficacy has affected the incidence of IPV during the pandemic. In particular, we examined the effect of individual-level collective efficacy, because it would have been costly and challenging to continuously measure neighborhood-level collective efficacy across several time points. As mentioned, however, whether measured at the neighborhood or individual level, collective efficacy has been found to reduce the incidence of IPV. Thus, we measured the latter: perceived collective efficacy at the individual level. Communities with high collective efficacy have social norms that do not condone violence [24] and are likely to intervene with victims [16]. As a result, victims in such neighborhoods have greater expectations of being interfered with by other members of the community and are more likely to seek support and outlets for self-disclosure [13], thereby making IPV less likely to escalate. Based on that evidence of collective efficacy’s protective effect against IPV, we predicted the following:

**Hypothesis** **1** **(H1).** *Collective efficacy established before the COVID-19 pandemic would reduce the incidence of IPV victimization during the pandemic*.

### 1.2. Association between Changes in Collective Efficacy and Changes in IPV

Our second task was to explore the relationship between changes in collective efficacy and changes in IPV during the COVID-19 pandemic. On that topic, several studies have shown a possible decrease in social capital during the pandemic. For example, in an analysis of a large representative sample of the German population, sociability and trust in society, both elements of social capital, declined in 2020–2021—that is, during the pandemic—compared with 2017–2018 before the public health crisis [25]. Another study comparing the degree of perceived social capital among youth in China has revealed that community social capital declined after the initial lockdown during the first wave of COVID-19 [26,27].

Thus, it is possible that collective efficacy, which is closely related to social capital [28,29], has also declined throughout the pandemic. By extension, if collective efficacy has decreased in the neighborhood where intimate partners live, then their IPV may have been more likely to escalate. As noted, partners who live in communities with higher collective efficacy are more likely to interact with neighbors whose social norms do not condone violence [24]. Such partners are more likely to expect intervention [16] and support from their neighbors as well as to self-disclose [13], thereby making IPV less likely to escalate. Taken together, if a neighborhood’s collective efficacy has decreased during the COVID-19 pandemic, then opportunities for supportive interactions with neighbors have been lost, and the incidence of IPV may have increased.

However, decreased collective efficacy during the pandemic may have also encouraged people to interact and maintain relationships with their neighbors, which may have suppressed IPV. For example, in a survey of 13 countries, including Turkey, the United Kingdom, and the United States [30], as well as a study in Spain [31], the more strongly people perceived collective efficacy among their neighbors, the more actively they distanced themselves socially. Social distancing from others may have also reduced social interactions with IPV-suppressing neighbors [13,16,24], thereby resulting in an increased risk of IPV in areas with higher collective efficacy. Conversely, the reduction in collective efficacy during the pandemic may have suppressed IPV by maintaining social interactions with IPV-suppressing neighbors.

Thus, changes in collective efficacy during the COVID-19 pandemic could be either positively or negatively correlated with the incidence of IPV. However, because no study, at least to our knowledge, has sought to determine which correlation is more plausible, we empirically examined the association between changes in those indicators in our study. We aimed to test our hypothesis and how changes in IPV correlate with changes in collective efficacy based on longitudinal data collected before and during the pandemic. Such panel data were expected to enable us to capture the impact of preexisting variables on subsequent variables over time and, in turn, to infer causal relationships. The data also allowed us to understand whether changes in each variable have been covariate.

### 1.3. Purpose of the Study

We analyzed data from a survey conducted at multiple time points in Japan before and during the COVID-19 pandemic to test the association of individual trajectories of IPV and collective efficacy with latent growth models (LGM). Our study addressed two research questions. First, by testing our hypothesis, we sought to determine whether collective efficacy’s suppressive effect on IPV has been sustained or reduced amid the unprecedented circumstances of the pandemic. Following reports that collective efficacy’s suppressive effect on IPV may be situation-dependent [18], we tested the generalizability of that effect during the pandemic.

Second, in an exploratory analysis, we sought to determine how changes in collective efficacy have been associated with changes in IPV after controlling for the effects of collective efficacy before the pandemic. Previous research on the association of collective efficacy and IPV has chiefly focused on variations across communities, meaning that few studies have probed the impact of changes in collective efficacy. An exception, however, has shown that flooding and cyclones in Australia reduced residents’ perceived collective efficacy [19]. By extension, the pandemic may have resulted in similar changes in collective efficacy [25,27,31], and exploring that potential outcome can contribute to a deeper understanding of the processes by which collective efficacy functions.

Last, given the characteristics of our data—as mentioned, collected at multiple time points before and during the COVID-19 pandemic in a sample of adults in Japan—we conducted a parallel LGM analysis to examine the effect of initial values of two or more changed variables on subsequent changes and the association between those changes [32]. We assumed that the pandemic has altered people’s social lives in Japan, that the changes have been more remarkable over time, and that LGM analysis was best suited to process the data.

## 2. Materials and Methods

Our study was approved by the Ethics Review Board of the Department of Management in the Graduate School of Social Sciences at Hiroshima University on 26 April 2019.

### 2.1. Procedure

We invited monitors of an online research firm with a romantic partner or spouse to participate in the survey. Willing monitors were briefed on the purpose, conditions, and ethical considerations of the study and consented to participate by responding to the survey via the external survey website Qualtrics. Recruitment and the screening survey were conducted from June to August 2019, and the first survey was performed in September (i.e., T1) before the COVID-19 pandemic. The survey was then administered 3 additional times (i.e., T2, T3, and T4) during the pandemic at approximately 7-month intervals. Participants received points from the online research firm for each response.

To detect *satisficing* [33,34], defined as behavior attempting to minimize the effort needed to complete a survey [35], we included a scale of directed questions in the screening survey, and only respondents who did not give a satisficing response were included in the main survey (i.e., 606 of 729). In each subsequent survey, an item was also included to detect satisficing. During the survey, respondents who did not adequately follow the instructions were automatically given a warning and reminded of the need for careful responses; they were subsequently asked to correct their answers and continue responding to the items. Last, from T1 to T4, only respondents who correctly answered the items to detect satisficing were eligible to proceed to the final screen.

In Japan, cases of COVID-19 infection began to be widely reported in March 2020, and the first state of emergency was declared in April 2020. The declaration was reissued intermittently on four subsequent occasions until the survey ended at T4. With each declaration, the public was asked to refrain from unnecessary outings (i.e., to practice social distancing). Therefore, we assumed that the survey data from T1 reflected the pre-pandemic situation, whereas the data from T2 onward reflected the situation after COVID-19 began to spread in Japan.

In parallel LGM analysis, we regressed the slope (i.e., change) in IPV or collective efficacy on the intercept (i.e., T1) of collective efficacy or IPV to test our hypothesis. Afterward, in an exploratory study, we tested the covariance of the slope (i.e., change) in IPV and collective efficacy scores.

### 2.2. Participants

The 318 participants who completed the survey at T1 along with at least one of the surveys from T2 to T4 and who remained in a relationship with the same partner at T1 throughout the survey period were included in the sample for analysis. Participants had a mean age of 32.87 years (*SD* = 4.66) at T1, and there were 157 men and 161 women. The average length of their relationships was 72.90 months (*SD* = 74.63), 61.6% of them were married, and 70.1% were living with their partners. There were 303 participants at T2, 248 participants at T3, and 227 participants at T4.

### 2.3. Measures

Participants were asked to respond to two scales. First, for collective efficacy [11], we used a scale developed in Japanese based on Sampson et al.’s scale [10]. As the original scale, our scale consisted of two factors: informal social control and social cohesion and trust. *Informal social control* referred to the tendency of neighbors to cooperate in dealing with neighborhood problems; its six items included, for instance, “I think that people in the community intervene and get involved in some way when there is a problem in the neighborhood.” By contrast, *social cohesion and trust* referred to a relationship of trust between neighbors; its six items included, for example, “People in the community trust each other.” 

Sampson et al. [10] examined collective efficacy by averaging the scores of two of its subfactors. However, recent studies [36,37] have indicated that those subfactors should be regarded as capturing different aspects of collective efficacy. The results of our confirmatory factor analysis also showed a distinction between the concepts; to be specific, the two-factor model (χ^2^ = 844.14, *df* = 66, *p* = 0.001, root mean square error of approximation [RMSEA] = 0.08, comparative fit index [CFI] = 0.96, Tucker–Lewis index [TLI] = 0.95, standardized root mean square residual [SRMR] = 0.06, Akaike’s information criterion [AIC] = 8765.62, Bayesian information criterion [BIC] = 8904.70) fit better with the data than the one-factor model (χ^2^ = 846.36, *df* = 54, *p* = 0.001, RMSEA = 0.22, CFI = 0.72, TLI = 0.65, SRMR = 0.16, AIC = 9438.89, BIC = 9574.21). We recorded model fit indices for the SEM, including the confirmatory factor analysis described above, following a previous study [38]; there were thus four approximate model fit indices (i.e., RMSEA, CFI, TLI, and SRMR) and two information criteria (i.e., AIC and BIC). With respect to the former index, models with an RMSEA less than 0.08, CFI and TLI values greater than 0.95, and an SRMR less than 0.08 have been evaluated to fit the data better [39]. For the latter, AIC and BIC, models with smaller values have been rated as fitting the data better than other models [40]. Therefore, we concluded that collective efficacy was composed of two subfactors and calculated the mean score for each, which we used in our subsequent analysis.

Second, to measure IPV suffering, we used a shortened version of the Conflict in Adolescent Dating Relationships Inventory (CADRI), which consists of five subscales: Physical Abuse, Threatening Behavior, Sexual Abuse, Relational Abuse, and Verbal and Emotional Abuse [41]. The first author and a counselor (i.e., not an author) fluent in English and involved in IPV prevention programs translated the original English CADRI into Japanese with the permission of the CADRI’s creators so that respondents could easily understand the items without losing the meaning of the original version. The translated version was back-translated using the translation software DeepL to ensure that the content of the English back-translation was generally equivalent to the original. In the original study, analyses on subscale-specific and overall scores were conducted; however, based on our confirmatory factor analysis of the data from T1, we used the overall mean of the items as the scale score instead. The model assuming one factor (χ^2^ = 78.11, *df* = 30, *p* = 0.001, RMSEA = 0.07, CFI = 0.98, TLI = 0.97, SRMR = 0.02, AIC = 3917.22, BIC = 4048.45) fit the data better than the model assuming five factors (χ^2^ = 556.86, *df* = 25, *p =* 0.001, RMSEA = 0.26, CFI = 0.79, TLI = 0.62, SRMR = 0.26, AIC = 4405.97, BIC = 4555.95). In the one-factor model, covariance was set between items on the subscale based on the semantic overlap of words in the items.

Although we also used other measures in our study, we do not report them here because they were irrelevant to our study’s purpose. Readers interested in those measures are welcome to contact the corresponding author.

### 2.4. Analysis

After identifying the mean values of each variable and the correlations among the variables, we used Mplus version 8.8 to conduct LGM analyses with structural equation modeling. All input files are available upon request. LGM analysis allows examining changes in a variable at three or more time points and how those changes relate to the variable at a given point. We also examined the model fit of the LGM using the fit indices as well as confirmatory factor analysis. In the presence of missing values, Mplus uses the full information maximum likelihood estimator [42]. We first fit three univariate LGMs to each dataset to describe changes in collective efficacy and IPV, respectively, which allowed us to understand how each variable has changed since before the COVID-19 pandemic. After that, parallel LGM analyses were performed. As mentioned, data for T1 were obtained before the pandemic, whereas data for T2, T3, and T4 were obtained during the pandemic. Because we generally fixed the intervals between the time points, T1 was used as the reference point, and the coefficients corresponding to each time point from T1 were set at fixed values (i.e., 0, 1, 2, and 3). In our analysis, we set the level of significance at 5%.

To test our hypothesis—that is, that collective efficacy established before the COVID-19 pandemic has reduced the incidence of IPV victimization during the pandemic—in parallel LGMs the change (i.e., slope) of IPV and collective efficacy were regressed on the intercepts of the other. That procedure allowed us to test our hypothesis while controlling for the reverse impact (i.e., from pre-pandemic IPV to subsequent changes in collective efficacy). We also estimated parameters of covariance between the changes (i.e., slopes) of IPV and collective efficacy in an exploratory examination. That follow-up procedure allowed us to address the second task of our study—that is, to test the linear association between changes in IPV and changes in collective efficacy during the pandemic.

## 3. Results

Means and correlations for the indicators are presented in Table 1.

### 3.1. Univariate LGM

To gain a descriptive understanding of how each variable changed during the COVID-19 pandemic, we performed a univariate LGM analysis three times for each variable. The slope estimates shown in Table 2 represent the level of linear change in each indicator. IPV (slope ipv = 0.00, p = 0.87) and social cohesion and trust (slope social cohesion and trust = −0.02, p = 0.24) showed no significant change from T1 to T4, whereas informal social control decreased significantly (slope informal social control = −0.08, *p* < 0.01). Moreover, as shown in Table 1, the man values were generally low for IPV (*M* = 1.22–1.32), a variable that may be regarded not as a continuous variable but as censored data. Therefore, we compared the goodness of fit in a censored model and in a censored-inflated one with that in the normal model. Both AIC and BIC values indicated that models with lower values fit the data better than others [40]. The results showed that the normal model (AIC *=* 1350.617, BIC *=* 1384.617) was a better fit than the censored (AIC *=* 1788.481, BIC *=* 1822.311) and censored-inflated model (AIC *=* 1672.421, BIC *=* 1725.046). We thus treated IPV as a continuous variable in subsequent analyses.

### 3.2. Parallel LGM

**Figure 1:**
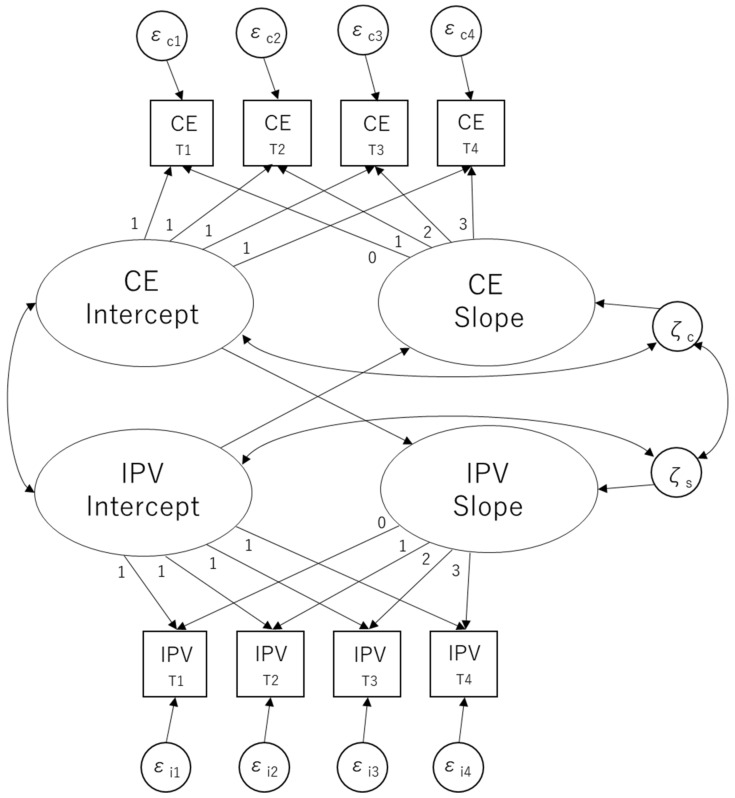
Parallel latent growth model. Note. CE = collective efficacy (i.e., informal social control or social cohesion and trust).

Two parallel LGM analyses were conducted to test the association between changes in the two variables (i.e., IPV and one of the subfactors of collective efficacy) in order to analyze the hypothesis and the exploratory examination simultaneously. To test our hypothesis, we regressed the slope of IPV and collective efficacy (i.e., informal social control or social cohesion and trust) on the intercept of the other. We also estimated the parameters of covariance between the slopes of the two variables and between the intercepts and slopes for the same variables, as shown in Figure 1.

Both the model including informal social control (χ^2^ = 51.86, *df* = 22, *p* = 0.001, RMSEA = 0.07, CFI = 0.92, TLI = 0.90, SRMR = 0.06) and the model including social cohesion and trust (χ^2^ = 49.40, *df* = 22, *p* = 0.001, RMSEA = 0.06, CFI = 0.95, TLI = 0.94, SRMR = 0.05) fit the data well.

As shown in Table 3, in Model 1 the informal social control intercept negatively affected the slope of IPV (γ = −0.05, *p* = 0.03). In Model 2, the intercept of social cohesion and trust also negatively affected the slope of IPV (γ = −0.05, *p* = 0.01). In other words, both pre-pandemic collective efficacy, whether informal social control or social cohesion and trust, suppressed subsequent IPV. Those results supported our hypothesis.

Model 1 revealed a significant positive association between the error term in the slope of informal social control and the error in the slope of IPV (*ψ =* 0.01, *p* < 0.01). Model 2 also showed a significant positive association between the error term in the slope of social cohesion and trust and the error in the slope of IPV (*ψ =* 0.01, *p* = 0.01). Those results indicate that the lower the collective efficacy during the pandemic (i.e., the lower the informal social control and social cohesion and trust), the lower the rate of IPV.

Last, among unexpected results, when initial IPV was greater, the slope for social cohesion and trust was smaller. The intercepts of IPV and social cohesion and trust were also positively associated, such that the greater the initial IPV, the greater the initial social cohesion and trust as well.

## 4. Discussion

We tested the hypothesis that collective efficacy prior to the COVID-19 pandemic has negatively affected changes in IPV during the pandemic, namely in Japan, as well as explored the association between changes in collective efficacy and IPV during the pandemic.

Before referring to the results of our analysis regarding our study’s main objective, we should highlight the results of the univariate LGM analyses conducted to describe the changes in each variable. As indicated by the slope in the top row of Table 2, univariate LGM analysis showed that collective efficacy decreased during the pandemic, whereas IPV levels did not change significantly. That finding is consistent with other findings from Japan. For instance, a nationally representative survey conducted every 3 years by the Gender Equality Bureau of the Cabinet Office also showed no increase in the number of reported cases of IPV in 2020 compared with 2014 and 2017, which partly reflects the impact of the COVID-19 epidemic in Japan [8]. In addition, the number of consultations with the Japanese National Police Agency did not increase much in 2020 or 2021 compared with 2019 [9]. Thus, no data currently available to us indicate an apparent increase in IPV in Japan during the pandemic.

As shown by the slope in the middle row of Table 2, however, our data do suggest that informal social control, one of the two aspects of collective efficacy, has declined since the beginning of the COVID-19 pandemic. For a possible explanation, once the state of emergency was declared in Japan, the government strongly urged the public to refrain from going out unnecessarily, thereby facilitating people’s restraint in leaving their homes. Such restraint may have reduced opportunities for contact with others and dampened the perception of informal social control in the community. Even so, the fact that the slope of the bottom row in Table 2 was not significant suggests that cohesion and trust were not affected by short-term fluctuations in opportunities for contact with others and may not have declined during the period of our study. Cohesiveness and trust may have resulted from residents’ accumulated evaluations of the trustworthiness of their neighborhoods over a more extended time. The implications of those and other results are described in what follows.

### 4.1. Effects of Pre-Pandemic Collective Efficacy on Changes in IPV

For both subscales of collective efficacy, the results of the parallel LGM analyses supported our hypothesis, as shown by the estimates in the second row of Table 3 representing Model 1 and Model 2. Coefficients regressing the slope of either collective efficacy or IPV on the intercept of the other indicated that collective efficacy before the COVID-19 pandemic suppressed the escalation of IPV during the pandemic. In the literature [13,17,21,22], the association between collective efficacy and IPV is reported based on cross-sectional data. By comparison, the results of our study are valuable in that they show that preexisting (i.e., pre-pandemic) collective efficacy influenced subsequent (i.e., mid-pandemic) IPV victimization. Moreover, our data show that prior collective efficacy had a suppressive effect on subsequent IPV even during the pandemic’s unprecedented circumstances, whereas other studies have shown that the IPV-suppressing effect of collective efficacy is situation-dependent [15,16]. We have thus demonstrated the generalizability of that outcome. Although no studies, at least to our knowledge, have examined collective efficacy’s suppressive effect on IPV in Japan, collective efficacy has been shown to suppress antisocial behavior, albeit not specifically IPV, in Japan as well as in China, South Korea, and the United States [12]. Our results indicate that collective efficacy serves to suppress IPV in Japan as well.

### 4.2. Relationship between Changes in Collective Efficacy and IPV

The correlations between the slopes shown in row 6 of Table 3 representing both Model 1 and Model 2—that is, the slopes of informal social control and social cohesion and trust, both of which constitute collective efficacy—were positively associated with the slope of IPV. Given the results of our univariate LGM showing that informal social control decreased during the spread of COVID-19, it seems that the more that the level of informal social control was maintained, the more likely it was that IPV increased. At the same time, the stronger the social cohesion and trust, the more likely it was that IPV increased as well. Beyond that, IPV tended to increase among individuals who perceived that neighborhood cohesion had increased and that informal social control had been maintained during the pandemic. A possible reason is that collective efficacy during the pandemic has reduced people’s opportunities for contact with others in their communities due to social distancing [30,31]. As a result, we speculate that, since the beginning of the pandemic, intimate partners have been less likely to receive normative interventions from others [24], to expect interventions from neighbors [16], to disclose themselves, and to receive support from others [13].

### 4.3. Strengths and Limitations

Our study contributes two major findings to the literature. First, we found a late effect of collective efficacy in preventing the escalation of IPV during the COVID-19 pandemic. Although most previous studies have shown an association between collective efficacy and IPV based on cross-sectional data, our repeated data revealed that, with reverse causality from initial IPV to changes in collective efficacy controlled for, initial collective efficacy influenced later IPV. Second, we observed a positive association between changes in collective efficacy and IPV during the pandemic. Although collective efficacy has traditionally been shown to be capable of reducing IPV [13,14], when it has functioned to promote social distancing in order to prevent the spread of COVID-19 [30,31], it has been associated with increased IPV victimization.

Those findings suggest that collective efficacy’s protective effect against IPV depends on the situation. As highlighted in previous studies, collective efficacy in the pre-pandemic promoted the interaction of community members and partners that resulted in a normative or supportive influence that consequently served to reduce IPV victimization. However, when social distancing was recommended to prevent the spread of COVID-19, collective efficacy inhibited that interaction and conversely increased IPV victimization. In other words, collective efficacy’s effect on IPV became moderated by whether opportunities for interaction with neighbors and partners were socially facilitated.

Our findings should be interpreted in light of our study’s limitations. For one, we did not directly measure changes in the amount of contact or the quality of interactions between partners and their neighborhoods. Although data suggest that collective efficacy promotes social distance [30,31], the ways in which that dynamic results in changes in interactions with others outside an intimate relationship remains unclear. Examining that relationship, however, would allow for a more detailed understanding of how collective efficacy suppresses IPV.

For another, we did not measure collective efficacy at the community level. It has been confirmed that both the individual- and community-level aggregate indexes of collective efficacy are negatively correlated with the incidence of IPV. Although using the perceived collective efficacy index allowed us to repeatedly measure changes across four time points, the scope of Sampson et al.’s study in which the index was created was the crime-suppressing effect of collective efficacy at the community level [10]. Future work should therefore confirm the variation between changes in IPV and collective efficacy at the community level.

## 5. Conclusions

Since the beginning of the COVID-19 pandemic, collective efficacy among neighbors may have promoted social distancing behavior and lost its preexisting capacity to suppress IPV. Using repeated data from four time points both before and during the pandemic in Japan, we tested the association between changes in collective efficacy and IPV at the individual level. Our results indicate that, as predicted, collective efficacy before the pandemic has had a protective effect on subsequent IPV. By contrast, changes in collective efficacy during the pandemic have been positively associated with changes in IPV victimization. In other words, sustained or increased collective efficacy during the pandemic may have increased the risk of IPV for partners in intimate relationships. We thus propose that the IPV-suppressing effect of collective efficacy depends on society’s encouragement of social interactions between neighbors and partners.

## Figures and Tables

**Table 1:** Means, standard deviations, reliability coefficients, and inter-correlations of variables for each time point.

		Mean	*SD*	*α*	1	2	3	4	5	6	7	8	9	10	11
1	IPV-T1	1.25	0.45	0.90											
2	IPV-T2	1.32	0.60	0.95	0.45 **										
3	IPV-T3	1.26	0.48	0.92	0.38 **	0.42 **									
4	IPV-T4	1.22	0.47	0.94	0.32 **	0.33 **	0.33 **								
5	Informal social control-T1	2.78	0.88	0.88	0.07	−0.08	−0.14 *	−0.14							
6	Informal social control-T2	2.62	0.92	0.92	0.09	0.15 *	0.16 *	0.14 *	0.43 **						
7	Informal social control-T3	2.49	0.98	0.91	0.01	−0.01	0.07	−0.08	0.33 **	0.43 **					
8	Informal social control-T4	2.53	0.94	0.89	−0.02	0.04	0.11	0.07	0.33 **	0.48 **	0.43 **				
9	Social cohesion and trust-T1	2.56	0.91	0.94	0.12 *	0.02	−0.08	−0.04	0.45 **	0.35 **	0.38 **	0.33 **			
10	Social cohesion and trust-T2	2.64	0.91	0.95	0.11	0.16 **	0.15 *	0.14 *	0.36 **	0.61 **	0.32 **	0.38 **	0.56 **		
11	Social cohesion and trust-T3	2.47	0.96	0.95	0.08	−0.04	0.05	−0.05	0.24 **	0.34 **	0.58 **	0.35 **	0.47 **	0.52 **	
12	Social cohesion and trust-T4	2.50	0.89	0.94	0.02	0.09	0.03	0.07	0.35 **	0.45 **	0.35 **	0.48 **	0.53 **	0.64 **	0.63 **

Note. IPV = intimate partner violence. ** *p* < 0.01. * *p* < 0.05.

**Table 2:** Parameters and goodness of fit for univariate LGM.

	Intercept	*SE*	*p*	Slope	*SE*	*p*	Correlation between intercept and slope	*p*	χ^2^	*df*	*p*	CFI	TLI	RMSEA	SRMR
IPV	1.28	0.03	0.00	0.00	0.01	0.87	−0.38	0.02	11.74	5.00	0.04	0.96	0.95	0.07	0.04
Informal social control	2.75	0.05	0.00	−0.08	0.02	0.00	−0.25	0.22	8.12	5.00	0.15	0.98	0.98	0.04	0.05
Social cohesion and trust	2.59	0.05	0.00	−0.02	0.02	0.24	−0.17	0.27	7.00	5.00	0.22	0.99	0.99	0.04	0.02

Note. Intercept and slope parameters are unstandardized estimates. CFI = comparative fit index; TLI = Tucker–Lewis index; RMSEA = root mean square error of approximation; SRMR = standardized root mean square residual.

**Table 3:** Parameter values of the parallel LGM.

					Estimates	*SE*	*p*
Model 1. Model for informal social control and IPV							
Intercept	IPV	→	Slope	Informal social control	−0.07	0.07	0.30
	Informal social control	→		IPV	−0.05	0.02	0.03
Intercept	IPV	↔	Intercept	Informal social control	0.04	0.02	0.10
Intercept	IPV	↔	Slope	IPV	−0.01	0.01	0.24
	Informal social control	↔		Informal social control	−0.03	0.03	0.30
Slope	IPV	↔	Slope	Informal social control	0.01	0.00	0.00
Model 2. Model for Social cohesion and trust and IPV							
Intercept	IPV	→	Slope	Social cohesion and trust	−0.13	0.06	0.04
	Social cohesion and trust	→		IPV	−0.05	0.02	0.01
Intercept	IPV	↔	Intercept	Social cohesion and trust	0.06	0.02	0.01
Intercept	IPV	↔	Slope	IPV	−0.01	0.01	0.29
	Social cohesion and trust	↔		Social cohesion and trust	−0.01	0.02	0.52
Slope	IPV	↔	Slope	Social cohesion and trust	0.01	0.00	0.01

Note. The estimated values of regression coefficients or covariance are unstandardized estimates.

## Data Availability

Data are available upon request.
